# Delivering postpartum family planning services in Nepal: are providers supportive?

**DOI:** 10.1186/s12913-018-3777-3

**Published:** 2018-12-06

**Authors:** Mahesh C. Puri, Manju Maharjan, Erin Pearson, Elina Pradhan, Yasaswi Dhungel, Aayush Khadka, Iqbal H. Shah

**Affiliations:** 1 Center for Research on Environment, Health and Population Activities (CREHPA), Kathmandu, Nepal; 2 Ipas, Chapel Hill, NC USA; 3 The World Bank Group, D, Washington, C USA; 4 Harvard T. H. Chan School of Public Health, Boston, MA USA

**Keywords:** Intra-uterine device (IUD), Service providers, Postpartum family planning, Supply-side barriers, Nepal

## Abstract

**Background:**

Health service providers play a key role in addressing women’s need for pregnancy prevention, especially during the postpartum period. Yet, in Nepal, little is known about their views on providing postpartum family planning (PPFP) services and postpartum contraceptive methods such as immediate postpartum intra-uterine devices (PPIUD). This paper explores the perspectives of different types of providers on PPFP including PPIUD, their confidence in providing PPFP services, and their willingness to share their knowledge and skills with colleagues after receiving PPFP and PPIUD training.

**Methods:**

In-depth interviews were conducted with 14 obstetricians/gynecologists and nurses from six tertiary level public hospitals in Nepal after they received PPFP and PPIUD training as part of an intervention aimed at integrating PPFP counseling and insertion into routine maternity care services. The interviews were audio recorded, transcribed, and analyzed using a thematic approach.

**Results:**

Providers identified several advantages of PPFP, supported the provision of such services, and were willing to transfer their newly acquired skills to colleagues in other facilities who had not received PPFP and PPIUD training. However, many providers identified several supply-side and training-related barriers to providing high quality PPFP services, such as, (i) lack of adequate human resources, particularly a FP counselor; (ii) work overload; (iii) lack of private space for counseling; (iv) lack of IUDs and information, education and counseling materials; and (v) lack of support from hospital management.

**Conclusions:**

Providers appeared to be motivated to deliver quality PPFP services and transfer their knowledge to colleagues but identified several barriers which prevented them from doing so. Future efforts to improve provision of quality PPFP services should address the barriers identified by providers.

**Electronic supplementary material:**

The online version of this article (10.1186/s12913-018-3777-3) contains supplementary material, which is available to authorized users.

## Background

Initiating contraceptive use during the immediate postpartum period is cost-effective and efficient from the perspective of both the health system and women, particularly in developing countries like Nepal where access to healthcare services remains low. Most Nepali women interact with the health system only during pregnancy, for delivery, or for immunizing their children. Providing family planning (FP) services during immunization visits is often difficult since immunizations are primarily done by dedicated health workers who are not trained in FP service delivery. In contrast, antenatal checks and deliveries are largely conducted by obstetricians, nurses, or auxiliary nurse midwives (ANMs). Given their background and conditional on receiving FP-related training, these healthcare providers are better suited to deliver FP services to Nepali women without adding the burden of recruiting new staff on the Nepali health system [1]. For Nepali women, provision of FP services in the immediate postpartum period allows them to conveniently meet their fertility goals as they do not have to make costly trips to health facilities at a later date for FP services.

Several studies across different settings have demonstrated the safety, effectiveness, and acceptability of immediate postpartum insertion of intrauterine devices (IUDs), especially when coupled with counseling on postpartum contraceptive methods [2–12]. Recognizing its many advantages, the Nepali government has focused its efforts on increasing the availability of long-acting FP methods and FP counseling services. Despite this, IUD use in Nepal remains very low: only 1.4% of currently married women in Nepal were estimated to be using an IUD in 2016 [13].

There are many reasons for the low uptake of IUDs in Nepal. First, unlike temporary FP methods which are available at most health posts, primary health care outreach clinics, or pharmacies, long acting reversible contraceptives (LARCs), such as IUDs, are only available at healthcare facilities that have obstetricians and gynecologists, or nurses, or ANMs who are trained in providing such FP services. In Nepal, both doctors and trained nurses are permitted to provide counseling and IUD insertion services. At the time of the launch of our study, a nurse or doctor attending a woman was expected to provide PPFP counseling or insertion services when requested to do so by the woman. However, there was no standardization in terms of the content and quality of the counseling provided to women and they largely depended on the workload faced by the service provider.

The effect of limited availability of LARCs on IUD uptake is compounded by the various geographical and financial barriers Nepali women face in accessing the health system. Additionally, medico-legal restrictions, fear of side-effects, poor perception of pregnancy risk, and socio-cultural factors such as lacking decision-making power due to lower status of women, pressure to give birth to at least one son, pressure to give birth soon after marriage, and stigma attached to pre-marital sex all affect the uptake of family planning in general and LARCs in particular [14–16].

Lack of knowledge or pre-existing biases toward FP services in healthcare providers may also reduce the uptake of postpartum family planning (PPFP) methods. A recent systematic review of studies from countries across six different continents showed that many providers have low levels of knowledge regarding IUDs and limited training in their insertion or removal [17]. Furthermore, many providers wrongly believe that IUD use results in serious side effects such as pelvic inflammatory disease and are thus reluctant to provide it to eligible women such as those who are HIV-positive [17].

In Nepal, providers’ views on PPFP and immediate postpartum IUDs (PPIUDs) in particular are seldom documented because of which the role of providers in uptake of these contraceptive methods is unclear. We also do not know if providers value effectiveness of a PPFP method over convenience or consider women’s preferences for a method and if such views differ by type of providers – doctors compared to nurses or ANMs. The degree to which women are involved in choosing a particular method may differ by type of provider as well as by the nature of the provider-client interaction. We aim to fill these gaps in the literature by exploring the perceptions and experiences of service providers in Nepal with regard to PPIUDs. We also seek to understand trained providers’ attitudes in sharing and diffusing their knowledge and skills about PPIUDs to other service providers who have not received specific training on PPFP or PPIUD counseling and insertion.

## Methods

### Parent study details

This study is nested within a broader trial studying the impact of an International Federation of Gynecology and Obstetrics (FIGO) led intervention to institutionalize PPIUD training and provision as part of antenatal and delivery services in six public, tertiary hospitals in Nepal. Hospitals were selected based on geographical location and high volume of obstetric cases (between 6000 and 11,000 a year). They were pair-randomized into two groups of three based on geographical location and annual obstetric caseload. Group 1 hospitals implemented the intervention after three complete months of baseline data collection while Group 2 hospitals implemented it after nine complete months of baseline data collection. The intervention was implemented by the Nepal Society of Obstetricians and Gynecologists (NESOG).

The intervention has three main components: 1) training healthcare providers on PPFP counseling including PPIUD and its insertion; 2) providing information, education and counseling materials; and 3) providing insertion supplies to study hospitals. The Nepal Health Training Center (NHTC) of the Ministry of Health and Population (MOHP) conducted the training in all study hospitals using a national protocol. Training was done over a course of three days and included both theoretical and practical components. Content areas covered in the training included healthy timing and spacing of pregnancy; various PPFP methods; overview on PPIUDs; counseling for PPFPs and, in particular, PPIUDs; client assessment for PPIUD; insertion and removal of postplacental postpartum transcaesarean IUD; infection prevention, side effects and complication management for PPIUD services; and, recording, reporting and tracking of clients. Counseling materials were adopted from the Global Library of Women’s Medicines (GLOWM) and were approved by the NHTC and National Health Education Information and Communication Center of MOHP. From a total of 146 providers working in the obstetrics and gynecology departments of the six study hospitals, 78 were trained as part of the intervention (30 obstetricians and 48 nurses/ANMs).

### In-depth interviews

We purposively selected 14 obstetricians/gynecologists and nurses from across the six hospitals and conducted in-depth interviews with them after they received training on PPFP and PPIUD as part of the intervention. We selected one physician and one nurse from five hospitals and two physicians and two nurses from the remaining hospital. Our decision to select 14 providers for in-depth interviews was informed by our past experience on the optimal number of respondents to capture the diversity of views and professional experiences. We conducted two more interviews in one hospital because we had insufficient information from the initial two interviews in this hospital. We conducted interviews with both physicians and nurses. None of the selected physicians and nurses were providing PPIUD services before the training. We conducted all interviews between December 2015 and October 2017, taking into account the stepped-wedge nature of the intervention roll-out. No provider approached for the interview refused.

We developed the in-depth interview guide in English (included as an Additional file 1) and translated it to Nepali. We did not back-translate the guide from Nepali to English; however, a third party not involved with the translation thoroughly checked the guide for accuracy. We covered a range of topics in the interviews including the background characteristics of respondents; knowledge, experiences, and preferences regarding contraceptive methods; experiences with PPIUD training and scale-up; and, their willingness to train others in their hospital and in other health facilities. We pre-tested the interview guide with four participants to assess question phrasing, question sequencing, and overall comprehension. Based on the results from the pre-test, we modified the interview guide.

### Interview protocol

At the start of each interview, participants were asked to review and sign an informed consent form. They were given the opportunity to ask any questions they had regarding the overall study as well as the interview. After obtaining signed informed consent, a trained researcher conducted the interview in a private room in each facility and in the Nepali language. The interviews were audio-recorded with permission from participants. These recordings were then transcribed and translated into English.

### Analytical strategy

We adopted a thematic approach to analyzing the data. First, we developed a codebook based on interview questions and an initial reading of interview transcripts. Two co-authors then independently read transcripts and developed the initial codes which were subsequently modified to develop a more refined codebook. The same co-authors coded all interviews using the codebook. Content codes were grouped into thematic categories. Key quotes that exemplified major themes and concepts are presented below.

ATLAS.ti (Version win 7.0, Scientific Software Development, GmbH, Berlin, Germany) was used for organizing the text, coding the data, grouping codes into relevant themes, and presenting the key themes.

### Ethical approval

This study was approved by the Ethical Review Board of the Nepal Health Research Council (NHRC).

## Results

### Participant profiles

Of the 14 participants interviewed, 7 were obstetricians and gynecologists, 5 were nurses, and 2 were auxiliary nurse midwives (ANMs). Most participants were female, between the ages of 21 and 52 years, and had one to 13 years of professional experience. On average, interviews lasted approximately 75 min (range: 55–97 min).

### Perspectives on postpartum family planning services and methods

Service providers recognized the importance of providing postpartum contraception and, in particular, immediate PPIUD. They highlighted two benefits of immediate PPIUD in particular: first, it is a long-term method of contraception; and second, it has fewer side effects such as not interfering with breastfeeding. One service provider said:

*“I take PPIUD in a positive way. Inserting it within 48 hours of delivery will not be that uneasy for women. It will be difficult for them after 6 weeks. Woman won’t go through pain if it is inserted immediately after delivery and also they don’t have to come again in the hospital for the service. This method is beneficial for mother, baby and family as a whole. This method has no hormone so it does not have side effect. It does not hamper breast milk and breastfeeding women. If inserted once it acts as a temporary method for 12 years. Due to all these factors, I have a positive attitude towards PPIUD”.*

– ID 10, nurse, less than 5 years of work experience.

Most service providers (11 out of 14) highlighted two additional benefits of providing FP services immediately after delivery: it addressed the missed opportunity of providing FP services to women and this, in turn, reduced unmet need for family planning. Specifically, they stressed on how women rarely come to health facilities just for FP services; however, they often come for deliveries which means that inserting PPIUD immediately after a delivery could help women save time and money.

*“Overall I am very positive towards PPIUD….there is still high unmet need of family planning. Many women are likely to be missed because family planning service are generally not provided in maternity ward. Women had to go to maternal and child health clinic to get family planning methods ….. …Only very few women come for the sole purpose of getting contraceptives. This is why when we provide immediate PPIUD service right at the time of delivery we can serve many women right on time.”*

– ID 02, nurse, less than 5 years of work experience.

*“Copper T - PPIUD, which we are initiating from our health institution. This is very best, if we are able to provide good counseling then this can be a very good method for women”.*

– ID 04, Obstetrician/gynecologist, 5–10 years of work experience

Although providers recognized the many benefits of PPIUDs, they also understood the importance of involving clients in the process of making recommendations about using PPIUDs. Many providers, including doctors, nurses, and ANMs, characterized their approach as “client driven” and suggested that they only make FP recommendations after talking with clients about their fertility goals and preferences. However, most service providers also stated that they use criteria such as a client’s age, parity, education, and breastfeeding status to determine which FP method would be most suitable.

*“There are many postpartum contraceptive methods… There are many factors to consider like, which methods to use, which not to use, which methods can be taken immediately and which can be taken later. It depends on the women’s age, the number of children they have, desire to have another child. I even need to consider whether they have any other disease.”*

– ID 05, Obstetrician/gynecologist, over 10 years of work experience.

*“If a woman is below 18 years and giving first birth then we don’t talk about permanent family planning methods with her. We provide detail information about temporary family planning methods with such women. If a woman 40 is years and comes for counseling about family planning methods then we ask them about the number of living children and counsel her in a different way”.*

– ID 10, nurse, less than 5 years of work experience

### Perspectives on the postpartum family planning training intervention

In general, providers found the content of the training intervention to be comprehensive and well presented. They were satisfied with the breadth and depth of information they received on various FP methods.

*“Overall, everything about the training was good. There was sufficient information on temporary as well as permanent FP methods. There were posters on FP and information on exclusive breastfeeding as a means of temporary family planning method was also provided”.*

– ID 02, nurse, less than 5 years of work experience.

In terms of learning about FP methods and service delivery, almost all providers (13 out of 14) found training materials such as demonstration models, posters, and brochures appropriate. Two providers explained:

*“In Nepal’s context, whatever things were required as per Family Health Division guideline, all those materials and methods were included in the training and it was sufficient.”*

– ID 04, obstetrician/gynecologist, 5–10 years of work experience.

*“They [trainers] had brought all the materials. They also brought posters on male and female permanent family planning methods. The materials and lectures were effective and impactful.”*

– ID 10, nurse, less than 5 year of work experience.

Twelve of the 14 interviewees found the training content on immediate PPIUD counseling, insertion, and management of complications to be especially useful. Many also noted the usefulness of dummy demonstration models (MAMA-U) for practicing PPIUD insertions to be helpful.

*“The most useful content of the training was correct ways of inserting IUD, eligibility criteria for PPIUD and type of equipment to use for insertion etc.… Without a dummy [MAMA-U], we wouldn’t have done that [PPIUD insertion]. We had a chance to learn the insertion technique. We learned what a speculum is like and how to use it, how to first hold it with a sponge holder and how to insert it. It was very easy to use the same method on the patient.”*

– ID 07, obstetrician/ gynecologist, 5–10 years of work experience.

Providers also appreciated the focus of the training intervention on transferring knowledge to other colleagues. One nurse, comparing their experiences in the training intervention to other trainings, said:

*“In previous training, we were only taught to give services but now in the training we were taught how to give service and also on how to train others and to bring into practice in other places as well. The main difference in this training is that we are able to transfer whatever knowledge and skills we have learned to other colleagues as well”.*

– ID 03, nurse, 5–10 years of experience.

However, participants also identified several aspects of the training that could have been more effectively organized. Six out of the 14 providers, mainly nurses, believed that the duration of the training should have been longer. They argued that the short duration of the training resulted in certain topics being missed and not enough time being allocated to practicing PPIUD insertions.

*“The training period should be extended to at least 5 days, trainee should be taken to client side and allowed to practice, and just providing training is not enough we should be updated time and again”.*

– ID 03, nurse, 5–10 years of work experience.

*“All the participants couldn’t practice [PPIUD insertions] properly due to limited time in the training. Participants would have gained more knowledge and experience if the training was for a longer duration. Training duration should be at least for 5 days to make it more effective.”*

– ID 09, obstetrician/gynecologist, 5–10 years of work experience.

*“[The trainers] tried to cover everything in [a] short period of time. I think they missed out on removal of PPIUD due to the short time.”*

– ID 14, obstetrician/ gynecologist, 5–10 years of work experiences.

Three service providers also thought that demonstrating PPIUD insertion using the MAMA-U dummy model was not sufficient. They were especially critical of the discrepancy in the number of dummy models to number of training session participants.

*“We (participants) were large in numbers and there were only 2 dummies for practice. So, everyone could not get enough time to practice”.*

– ID 12, obstetrician/ gynecologist, less than 5 years of work experience.

### Perspectives on providing postpartum family planning services

Almost all providers (13 out of 14) expressed confidence in providing PPFP services including PPIUD insertion, complication management, and removal. They felt well prepared to answer questions and respond to concerns that women may have regarding PPIUD.

*“When we are counseling, they [patients] ask us if anything will happen because of it [PPIUD]. They ask us what else will happen. We need to able to answer to all of their questions. I think, after this training, I am able to answer all of the questions.”*

– ID 01, obstetrician/gynecologist, 5–10 years of work experience.

*“I was not confident to insert PPIUD before, after getting this training, I develop my confidence.”*

– ID 02, nurse, less than 5 years of work experience.

However, despite receiving training, some nurses and ANMs in particular still believed that they needed additional support from their senior colleagues on managing PPIUD-related complications. They also recognized the critical importance of regularly scheduled refresher trainings and increasing the number of staff available to provide FP services. For example, one nurse noted:

*“If there is any complication while inserting IUD, I need to inform our doctors. Only after informing the doctors, we try to manage complications. We are not fully confident in managing complications…Refresher training about this should be given from time to time and IUCD need to be made available on time…Trained manpower need to be increased. Also, all the staffs from labor and antenatal care ward should be given training.”*

– ID 03, nurse, 5–10 years of work experience.

Also, providers argued that the quality of PPFP services was compromised by several supply-side challenges. In particular, they highlighted the importance of maintaining a continuous supply of IEC materials, IUDs, and IUD insertion sets as well.

*“For full implementation of PPIUD service, we will require set (Kelly Forceps). The sets we have right now are not sufficient. If every client wants PPIUD then we don’t have enough supplies. We also need to increase trained manpower. If all these factors are met then we can fully implement the PPIUD services - counseling, insertion and removal.”*

– ID 02, nurse, 30–35 years old, less than 5 years of work experience.

*“We are ready to provide this service…The only thing is there should be regular supply of necessary IEC materials and IUD.”*

– ID 05, obstetrician/ gynecologist, over 10 years of work experience.

A few service providers also expressed doubts about their facility’s ability to institutionalize PPFP services. In particular, they argued that an insufficient number of staff and a high workload would prevent them from allocating the time necessary for PPFP services such as counseling. They were also skeptical about the sustainability of PPIUD services in their facilities after the current intervention was phased out.

*“As we have other works, we are not being able to provide adequate time in counseling. If we don’t have time and cannot provide counseling then the patient will not be able to know about the availability of PPIUD service”.*

– ID 11, nurse, 5–10 years of work experience

*“If NESOG [training provider] stops supporting the program, and if in case our own government doesn’t support this program (PPIUD) then there may be a problem of its continuation”.*

– ID 03, nurse, 5–10 years of work experience.

Finally, most participants (11 out 14) also expressed that the lack of a dedicated FP counselor and private space for counseling at their facilities could hinder the provision of PPFP services.

*“We do not have a separate counselor. Due to unavailability of a counselor, women are not receiving counseling which they have to receive…... When a separate counselor is there, the service can be easy and fast as well”.*

– ID 09, obstetrician/ gynecologist, 5–10 years of work experience

*“To make this service more effective, a trained counselor is essential at ANC [antenatal care] OPD [outpatient department] and that counselor should give adequate time in counseling. There should be a separate room at ward for the service and staff should be increased to increase the service”.*

– ID 13, nurse, less than 5 years of work experience

### Perspectives on increasing access to quality postpartum family planning service delivery

Participants provided many suggestions on increasing access to quality PPFP services in their facilities. Wary of the lack of staff and high workload, almost all providers suggested that having a dedicated counselor and a private counseling space would greatly improve PPFP service delivery in their hospitals.

*“We have higher case load and do not have adequate equipment. There are less manpower and do not have a trained counselor. There is a lack of separate of counseling room”.*

– ID 13, nurse, less than 5 years of work experience

*“Lack of time and space to provide the service is an obstacle. We are not in a position to provide individual counseling. We have less manpower and higher caseloads... The room is small and there is no adequate manpower. Both these can be solved by increasing the space and service providers….. The number of counselors should be increased for proper and effective counseling”.*

– ID 14, obstetrician/ gynecologist, 5–10 years of work experience

In addition, providers stressed on the need to solve supply-related challenges, in particular the lack of necessary equipment to provide PPIUD insertion and removal services. They also suggested that greater involvement from concerned government departments in terms of monitoring could improve the quality of services being offered at their facilities.

*“The Family Health Division should do supervision and monitoring on a regular basis, the hospital should make this [PPIUD] service as a general regular service, all the necessary materials should be supplied on time to make the service better and sustainable.”*

ID 05, obstetrician/ gynecologist, over 10 years of work experience

*“The only thing is there should be regular supply of necessary materials and IUCD as we had to discontinue this service before because of lack of IUCD”.*

– ID 05, obstetrician/ gynecologist, over 10 years of work experience.

### Perspectives on transferring knowledge and skills to other providers

All providers were willing to transfer their knowledge and skills regarding PPFP and PPIUD to their colleagues in other facilities. They also expressed their desire to continue providing services in the private sector.

*“..Yes [transferring knowledge and skills] is definitely possible. There is no new thing to be added…Infection prevention is same thing and the copper T is also same thing. The technique of inserting the copper T at this health institution and other health institutions is the same. It does not matter, it can be inserted in other health institutions…….It can be done in the private. The service provider here also goes to private to provide service so there is not any difficulty in transferring skills other providers”.*

– ID 04, obstetrician/ gynecologist, 5–10 years of work experience

*“If I get transferred to another health facility or work in the private sector and if women come in need of this service and if there are adequate materials, I can provide the service. For this, health facility should have adequate rooms, a PPIUD set for the service. If the health facility can arrange these necessary materials, then it will not be difficult for me to provide service on PPIUD”.*

– ID 13, nurse, less than 5 years of work experience.

However, providers also identified important barriers to ensuring the proper transfer of PPFP knowledge and skills. They argued that there was often little to no support from heads of hospitals and senior staff members to ensure knowledge and skills transfer. Furthermore, supply-side barriers in other facilities also complicated the process of transferring knowledge to providers within and outside of their facilities.

*“[The transfer of knowledge and skills about PPIUD] will depend upon the management of health facilities. If the health facility manager can manage necessary supplies then the service can be effectively provided. If such health facility considers PPIUD as an important method like any other FP methods then service delivery is possible. But, sometime private and government health centers may lack necessary manpower, materials, private hospitals may charge fee for service delivery, there may be less trained staffs, clients may show unwillingness to pay and get the service. If the health facility manages these issues then I have no problem in providing service.”*

– ID 09, obstetrician/ gynecologist, 5–10 years of work experience.

*“…If there are no [supplies] then it will act as a barrier [for transferring knowledge about PPIUD]. In case there is no PPIUD set then there will be a barrier for transferring skills … Sometimes when I try to insert PPIUD I do not get PPIUD, if there is no regular supply then it will also act as a barrier in a new place.”*

– ID 03, nurse, 5–10 years of work experience.

– ID 02, nurse, less than 5 years of work experience.

## Discussion

The in-depth interviews with 14 providers reveal that they view PPFP positively, that the training they received as part of the intervention was useful in many ways, and that they are willing to transfer their skills and knowledge to colleagues across different types of facilities. However, participants also identified several factors which could improve the quality of PPFP training, PPFP service provision, and the process of transferring knowledge and skills.

Barriers to quality service provision and diffusion of knowledge and skills were largely related to failures in the health system and did not revolve around a provider’s personal beliefs toward FP methods. Doctors, nurses, and ANMs all agreed that the lack of a dedicated FP counselor and private counseling space in a facility, irregular and insufficient supply of IUDs and IEC materials, insufficient number of staff, high workload, and lack of support from hospital leadership were key barriers to the provision of high quality FP services and transfer of knowledge and skills.

These barriers are consistent with previous research from Iran and Uganda which show that facility logistics impact contraceptive counseling and provision [18, 19]. Though postpartum contraceptive services, especially those that can be provided at the time of delivery like PPIUD, are generally thought to be easily integrated with maternity care [1], our findings suggest that integrating such services into the Nepali health system may not be feasible without addressing several supply-side challenges identified by the participants of this study.

Providers saw postpartum contraceptive services as a worthwhile endeavor as we found no negative attitude toward provision of PPIUD services. However, the need for a dedicated counselor for postpartum contraception suggests that there is insufficient time during antenatal care (ANC) visits to cover these topics despite providers’ willingness to do so. A recent study showed that primary care providers in Nepal spend an average of two minutes with each patient [20]. In one of our study hospitals, more than 200 women were served daily by only four providers for ANC check-ups. If service integration that complements the existing health structure is the goal, the Nepali health system should first focus on increasing existing human resources for health before scaling-up integrated postpartum contraceptive services.

Traditionally, the role of PPFP counseling has been largely assigned to nurses and ANMs while IUD insertions are done by doctors. In Nepal, however, doctors as well as trained nurses and ANMs are allowed to provide counseling and IUD insertion services. This practice of allowing different types of providers to deliver both counseling and insertion services is seen in neighboring India as well, is evidence-based [21, 22], and is recommended by the World Health Organization [23]. However, given their different training and roles within the healthcare system, it is natural to assume that nurses, ANMs, and doctors may have different notions about PPFP. We found that doctors, nurses, and ANMs agreed on a number of different issues surrounding PPFP, the training intervention, and in terms of disseminating knowledge and skills to colleagues. However, we also found that nurses and ANMS mentioned the need for additional refresher trainings and greater support from senior colleagues when handling PPIUD-related complications more often than doctors. Similarly, nurses and ANMs were also more likely to express concern about the short duration of the PPFP and PPIUD training relative to doctors. These results suggest that integrating PPFP training, counseling, and insertion services into the Nepali healthcare system will require not just addressing supply-side challenges but also ensuring buy-in from health facility management and potentially designing training sessions differently based on provider type.

Our results are subject to some limitations. First, given the qualitative nature of this study, we cannot generalize our results to all training participants. While broad themes may be applicable to other sites and providers, our results could also reflect issues and viewpoints specific to large public facilities in Nepal. Second, we interviewed providers immediately after they had received training on PPFP. This could have encouraged participants to talk more positively about the training they had received and the importance of immediate PPIUD services. Our study does not include information or perspectives of providers who were not trained by NESOG as part of the PPIUD intervention.

## Conclusions

The in-depth interviews with providers from six study hospitals highlight strengths and shortcomings of improving postpartum contraceptive provision from the perspectives of service providers. Providers were highly motivated to provide quality family planning FP services and transfer their knowledge and skills; however, they also identified several facility and training related factors which could prevent them from doing so. Interventions aimed at addressing structural factors (such as increasing human resources for health and private space for counseling) and other supply-side factors (such as timely and regular supply of the required equipment, refresher training, and regular monitoring and support visits from concerned government departments) are needed to improve postpartum family planning services in Nepal. Integrating postpartum family planning services into the healthcare system will also require ensuring buy-in from facility management and potentially training doctors, nurses, and auxiliary nurse midwives differently.

## Additional file

Additional file 1: Interview Guide with Service Providers after training for PPIUD. (DOCX 28 kb)

## Abbreviations

FIGO: International Federation of Gynecology and Obstetrics
FP: Family planning
IUD: Intra-uterine device
NESOG: Nepal Society of Obstetricians and Gynecologists
PPFP: Postpartum family planning
PPIUD: Postpartum intra-uterine device
