# Supplementary material for: Delivering postpartum family planning services in Nepal: are providers supportive?
Source: BMC Health Serv Res. 2018 Dec 6;18:948. doi: 10.1186/s12913-018-3777-3 (PMC6282334; doi:10.1186/s12913-018-3777-3)
Supplement: Supplementary file 1 — Interview Guide with Service Providers after training for PPIUD. (DOCX 28 kb) [file 12913_2018_3777_MOESM1_ESM.docx]

**Studying the impact and performance of institutionalizing immediate post-partum IUD (PPIUD) services as a routine part of antenatal counselling and delivery room services in Nepal**

**In-depth Interview with Service Providers after training for PPIUD**

***General Interview Guidelines:***

The topics and questions below should be used to guide the interview but can be adapted as necessary to each interview. Keep in mind when conducting the interview to respond to the answers provided by the respondent by asking additional questions to those proposed below or adapting to more appropriate questions. Suggested probes are listed below each question, but add your own probes as needed to ensure that the information provided is clear and to get more detail on topics mentioned that may be of interest to the study.

In particular, there are three main approaches to eliciting more information from the respondent:

1. Seek more detail or explanation of a response. For example:
   1. Tell me more about ______
   2. Can you give an example of _____?
   3. What happened next?
2. Explore the reasons behind a response. For example:
   1. What makes you say that?
   2. What was it about ____that made you decide to_____?
3. Seek clarity and check for inconsistencies. For example:
   1. Can you explain what you mean by ______?
   2. Earlier you said______ but it also seems like______. Can you explain?

The questions below should stimulate a natural flow of conversation, and therefore, the order of the questions can be altered, although you should go through each section in turn. Use the probe questions provided if that issue has not been spontaneously mentioned by the participant.

**Introduction (guidelines for the Interviewer)**

- Thank the participant for his/her time.
- Ensure you are in a private location where no one can overhear the conversation.
- Administer the informed consent procedure and ask the participant to sign the form if he/she is willing to be interviewed.
  - The participant should be provided the opportunity to ask any questions.
- Before starting the interview, please explain to the participant: ***Please note that we are interested in knowing your opinions and experiences and, therefore, please be frank in sharing these with us. There are no right or wrong answers, but your frank opinions and actual experiences are important.***
- Turn on the tape recorder, if respondent has no objection, before starting the interview.

1. **Knowledge, Experiences and Preferences for Contraceptive Methods**

*Thank you for taking time to talk with me.*

1. Could you describe any antenatal counseling you provide to women, if any?
   1. *Probe: What kinds of things do you talk about?*
   2. *Probe: Could you tell me about any information, if any, you provide related to:*
      1. *delivery?*
      2. *postnatal care?*
      3. *contraceptive use after delivery?*
      4. *postpartum IUD (PPIUD)?*
   3. *Probe: How much time do you typically spend with each client? How do you feel about the amount of time you spend with each client?*
   4. *Probe: How does the amount of time you have with a patient influence what you talk about? Which information do you focus on when you have less time?*
2. Tell me about your thoughts on postpartum contraception.
   1. *Probe: Describe how you see the relationship between breastfeeding and using contraception, if any.*
   2. *Probe: Which methods do you recommend, if any? Why do you recommend this/these methods? OR Why do you not recommend postpartum contraception?*
   3. *Probe: How can postpartum contraceptive counseling and methods best be provided to women (e.g. when, where)?*
3. What, if anything, do you think women do to prevent or delay pregnancy after delivery?
   1. *Probe: How do you think women view postpartum contraception? What do you think drives their interest/lack of interest?*
   2. *Probe: What role, if any, do you see family members playing in postpartum contraceptive decision-making?*
   3. *Probe: What methods do you observe women wanting to use during the six months following delivery? Why do you think they prefer these methods?*
   4. *Probe: How do you think women view the PPIUD?*
4. What influences the way you counsel on and provide postpartum contraception to women?
   1. *Probe: How do you approach counseling and providing contraception for women who come from different backgrounds?*
   2. *Probe: Describe how, if at all, you adjust your recommendations regarding postpartum contraception for different women.*
      1. *How, if at all, do you adjust your recommendations based on:*
         1. *Woman’s age*
         2. *Socioeconomic status*
         3. *Marital status*
         4. *Number of children*
         5. *Desire for more children*
         6. *History of contraceptive use*
5. **PPIUD Training Experience**

*Thank you. Now I would like to ask about your experiences with the NESOG training.*

1. Overall, what did you think about the postpartum contraception training from NESOG?
   1. *Probe: Which topics were most useful? Which topics were least useful?*
   2. *Probe: How did you feel this training related to previous training you received? (learned something new, was redundant, etc.)*
   3. *Probe: What could be improved about the training?*
2. Tell me about your thoughts on the training you received on postpartum contraceptive counseling.
   1. *Probe: How did you feel about the amount of training time spent on counseling (the right amount, too much, too little)?*
   2. *Probe: How prepared do you feel to manage:*
      1. *questions and concerns about PPIUD before insertion?*
      2. *questions and concerns about PPIUD after insertion, including requests for removal?*
      3. *questions and concerns about other methods?*
3. Tell me about your thoughts on the training you received on postpartum contraceptive provision.
   1. *Probe: What did you think about the number of methods covered? (the right amount, too few, too many)*
   2. *Probe: How prepared do you feel to manage:*
      1. *PPIUD insertion?*
      2. *PPIUD complications?*
      3. *PPIUD removal?*
   3. *Probe: On what topics, if any, would you have liked additional training?*
   4. *Probe: What did you think about the training materials (e.g. MAMA-U demonstration model)?*
      1. *Were they helpful/not helpful?*
      2. *What made them helpful/not helpful?*
4. How, if at all, do you expect your practice to change as a result of the training?
   1. *Probe: Could you tell me about any changes you anticipate in postpartum contraceptive:*
      1. *counseling*
      2. *recommendations*
      3. *PPIUD provision*
5. **Implementation, Scale-up and Diffusion of PPIUD Services**
6. Tell me about implementation of PPIUD services in your hospital.
   1. *Probe: What are the factors that could facilitate the full implementation of PPIUD services?*
      1. *How can these be put into place?*
   2. *Probe: What are the factors that could act as barriers to full implementation of PPIUD services?*
      1. *How can these barriers be overcome?*
      2. *Do you think the hospital will overcome these barriers?*
7. Based on your knowledge and experience, how can PPIUD services most effectively be expanded to other hospitals and providers?
   1. *Probe: How feasible would it be for trained providers such as yourself to provide PPIUD training to others?*
   2. *Probe: How feasible would it be for the government to equip other hospitals with the necessary supplies (e.g. forceps, IUDs, etc.)?*
8. How feasible would it be for you to provide PPIUD services in your private practice or if you moved to another hospital?
   1. *Probe: What are the factors that could act as barriers to implementation of PPIUD services in another hospital or private practice?*
      1. *How could these barriers be overcome?*
9. What are your overall impressions of PPIUD?
   1. *Probe: To what extent do you think PPIUD could be positive for women?*
   2. *Probe: To what extent do you think PPIUD could be negative for women?*
10. What would you want to add regarding your experience of training or opinions on postpartum contraception and PPIUD?

**Interview #___________________**

**Hospital _____________________**

1. **Background Information**

*Finally, I would like to ask a few questions about your background.*

1. **FROM OBSERVATION** CHECK THE APPROPRIATE OPTION:

- Male
- Female

1. How old did you turn on your last birthday? __/__ years
2. What is your professional background? ________________________
3. How long have been working in your current profession?

__/__ years [ENTER “00” IF LESS THAN 1 YEAR]

1. How long have you been providing family planning information or services?

__/__ years [ENTER “00” IF LESS THAN 1 YEAR]
